# Detection of Hepatitis C Virus Coinfection in Patients with Dengue Diagnosis

**DOI:** 10.1155/2014/321286

**Published:** 2014-05-14

**Authors:** Carlos Machain-Williams, Lourdes Talavera-Aguilar, Rosa Carmina Cetina-Trejo, Jaquelin Carrillo-Navarrete, Nubia Rivero-Cárdenas, Ma. Isabel Salazar, José Arturo Farfán-Ale, Fernando I. Puerto, María Eugenia Castro-Mussot

**Affiliations:** ^1^Laboratorio de Arbovirología, Centro de Investigaciones Regionales Dr. Hideyo Noguchi (Unidad Inalámbrica), Universidad Autónoma de Yucatán, 97225 Mérida, YUC, Mexico; ^2^Laboratorio de Enfermedades Emergentes y Re-emergentes, Centro de Investigaciones Regionales Dr. Hideyo Noguchi (Unidad Itzáes), Universidad Autónoma de Yucatán, 97000 Mérida, YUC, Mexico; ^3^Laboratorio de Inmunología Celular e Inmunopatogénesis, Departamento de Inmunología, Escuela Nacional de Ciencias Biológicas, Instituto Politécnico Nacional, 11340 Mexico, DF, Mexico

## Abstract

Coinfection produced by dengue virus (DENV) and hepatitis C virus (HCV) is a serious problem of public health in Mexico, as they both circulate in tropical zones and may lead to masking or complicating symptoms. In this research, we detected active coinfected patients by HCV residing in the endemic city of Mérida, Yucatán, Mexico, with positive diagnosis to dengue during the acute phase. We performed a retrospective analysis of 240 serum samples from dengue patients. The IgM-ELISA serological test was used for dengue diagnosis, as well as viral isolation to confirm infection. DENV and HCV were detected by RT-PCR. Thus, 31 (12.9%) samples showed DENV-HCV coinfection, but interestingly the highest frequency of coinfection cases was found in male patients presenting hemorrhagic dengue in 19/31 (61.29%), with a predominance of 12 : 7 in males. Firstly, coinfection of DENV-HCV in Mérida, Mexico, was detected in young dengue patients, between 11 and 20 years old (38.7%), followed by those between 21 and 30 years old (32%); only 16.13% were between 0 and 10 years of age. Diagnosis of HCV infection in patients with dengue is highly recommended in order to establish potential risk in clinical manifestations as well as dictate patients' special care.

## 1. Introduction

Worldwide, dengue virus (DENV) is one of the most important vector transmitted viruses. There are four DENV serotypes, classified as DENV 1, 2, 3, and 4, that cause similar clinical outcomes. Dengue is endemic in 112 countries, due in part to the increased geographical distribution of vectors in recent years [1].

Dengue fever (DF) and dengue with hemorrhagic manifestation (HD) are recognized as the world's second most prevalent infection in tropical and subtropical regions, after malaria, but the first in the American continent [2]. Infection with any of the serotypes can be accompanied by a fever, with increased vascular permeability and thrombocytopenia among other signs, but also dengue infection could turn out as unapparent.

Higher risk clinical manifestations, or severe forms, are associated with DENV serotypes 2 and 3, as in some cases dengue fever with HD or dengue shock syndrome has been associated to those serotypes [3, 4]. Globally, it has been estimated that nearly 100 million people per year suffer DF, and 500,000 HD, having a mortality rate of 0.5 to 3.5% [5]. In Mexico, the states with higher incidence are Morelos, Quintana Roo, Tabasco, Veracruz, and Yucatán [6], where the prevalence is estimated in 80% [7].

The circulation of other pathogenic agents infecting dengue patients has been detected, exacerbating the dengue clinical symptoms or confusing the diagnosis [8–10]. In tropical areas, concurrent infections with two different dengue serotypes, in the same patient, have been reported [11], besides coinfections by other arboviruses [12]. Also, in tropical zones, simultaneous circulation of several arboviruses and hepatitis C viruses (HCV) has been detected [13].

HCV is classified in the* Flaviviridae *family as a unique member of* Hepacivirus* genus. 80 to 85% of the cases of infection by HCV are chronic and responsible for hepatic injury. It has been estimated that HCV infects 3 to 4 million people each year. This means that nearly 170 million are at risk of a hepatic cirrhosis and hepatocellular carcinoma [14, 15]. HCV infection can be acquired in several ways, most regularly by exposure to contaminated blood or its products, maternal-fetal transmission, and in a lower rate sexual contact with infected people [16]. In 2011, Mexico reported that seroprevalence was 1.5% in general population, although among seropositive subjects only 48.3% presented HCV RNA [17].

HCV chronic infection is frequently associated with a marked thrombocytopenia [18], with the probable contribution of antiplatelet antibodies and the consequent alteration of the coagulation system [19]. Also, HD produced by DENV infection is characterized by thrombocytopenia associated with hemorrhage [20]. Here, we establish frequency of coinfections and examined the correlation of chronic HCV infection with severe dengue. The importance of coinfections produced by both viruses in public health conducted to this research, so we determined the presence of HCV in serum from patients with dengue positive diagnosis, residing in Mérida city, Yucatán.

## 2. Material and Methods

### 2.1. Patient Serum Samples

Sera samples were obtained from positive dengue patients in the acute phase of infection during the dengue epidemical outbreaks of years 2006 to 2008. They were collected at the arbovirology laboratory from Centro de Investigaciones Regionales Dr. Hideyo Noguchi, Universidad Autónoma de Yucatán (UADY). This protocol was approved by the UADY Bioethical Committee and every patient signed their authorization to participate in this investigation, as an informed consent. This was a retrospective, observational, and descriptive study. In total, 240 positive serum samples for dengue infection were analyzed, 120 (50%) from patients with HD and 120 from DF patients, abiding to the World Health Organization [4]. The basic epidemiologic information of patients included gender, age, and the first sign or fever date. Patients were not asked about HCV infection status.

### 2.2. Detection of DENV Infection

Diagnosis of DENV infection was performed by IgM detection with the MAC-ELISA test, previously described [21]. Also, molecular diagnosis was done by RT-polymerase chain reaction (PCR) test, using specific primers for viral genome [22] as shown in Table 1.

Amplicons were detected in 2% agarose gels stained with ethidium bromide and visualized in a transilluminator with ultraviolet light. As confirmation method, viral isolation was practiced using patient serum samples directly in Vero cells (green monkey kidney cells).

### 2.3. Detection of HCV Infection

The presence of anti-HCV IgG antibodies was looked for in all of the 240 serum samples from dengue positive patients, using the commercial kit Advanced Quality Rapid ANTIHCV Test (Accutrack, Xiamen, China). Also, detection of HCV RNA was done by a two-stage PCR with two pairs of primers deduced from the 5′-noncoding region [23] (Table 1).

### 2.4. Control Determination of VHC and DENV

As both viruses belong to the same family, primers were tested for crossed amplification using RNA extracted from peripheral blood mononuclear cells from DENV infected cells and positive controls for HCV to avoid amplification of false positive during PCR. HCV positive samples were obtained from Laboratorio de Enfermedades Emergentes y Reemergentes del Centro de Investigaciones Regionales Dr. Hideyo Noguchi, UADY. Viral RNA was extracted by PCR amplification tests with specific primers.

### 2.5. Statistical Analysis

Data was analyzed using contingency tables of chi-squared and* t*-Student calculations. Risk analysis for groups were done, considering relative risk >1. This was a cross-sectional study. Calculations were done according to program PASW 18.

## 3. Results

We identified the presence of HCV in 12.9% (31/240) of the examined dengue patient samples. From the 31 DENV-HCV coinfected patients analyzed here, 16 were males and 15 female patients, which indicate a similar distribution by gender, 15/109 (13.8%) in females and 16/131 (12.1%) in males; it does not show a significant statistical difference (Table 2).

Among the 16 coinfected male patients, HD was predominant in 12 cases, while only 4 had DF (Table 2). Analyzing the most associated dengue form with HCV coinfection, we found that the bigger HCV infection percentage (61.29%, 19/31) was in HD patients, while only 12/31 (38.71%) was in DF cases (Table 2).

In this study, we found that the age group that presented greater coinfection frequency was that of young patients, between 11 and 20 years with 38.7% of cases, being 7/12 males and 5 females. Second, 10 out of 31 coinfected patients (32%) were between 21 and 30 years with 5 cases from each sex. The group from 0 to 10 years of age included 16.13% of the total of coinfected cases that were distributed 3 : 2 (girls : boys). The less affected patient group by coinfection dengue-HCV were adults from 30 years and older (Table 3). The relative risk of presenting HD was 1.60 bigger for males with HCV infection with respect to females. Although the dengue patients were between 2 months and 68 years of age, with a mean of 15 ± 14 years, coinfection DENV-HCV cases were mainly presented in patients between 10 and 47 years of age (mean 21 ± 9) for males and 20 ± 12 (from 11 months to 46 years) for females (Table 3).

## 4. Discussion

The seroprevalence of HCV infection has been reported in 1.5 to 3.5% in Mexico and Latin America [14, 17]. Therefore, the frequency of HCV coinfection in dengue patients found here clearly indicates an increase over the current estimation, as it was almost 13%. This suggests that some conditions may contribute to an increased susceptibility of hepatic damage in coinfected patients, complicating the symptoms of either dengue or hepatitis C infection.

Although a bigger frequency of active HCV infection cases in patients with HD was observed, there was no statistical significance indicating a relationship between HCV infection and hemorrhagic manifestations during DENV infection, probably due to the sample size. Serologically, only one patient out of the 240 (0.4%) showed IgG antibodies to HCV.

The distribution of DENV-HCV coinfections found here indicates a predominance in HD male patients (12 : 7 M : F), as our original sample included 50%, 120, cases of DF and 50% of HD (Table 2). Besides, in the group of 16 sera from male patients that presented coinfection of DENV-HCV, 75% (12/16) corresponded to HD cases (Table 2). Despite the reduced sample size used in this research, a bigger association to double viral infection was evident in HD patients among all dengue male patients. These results are relevant considering that, worldwide, there are around 100 million of DF cases by 500 000 of HD [5].

Data indicates the necessity of identifying if DENV infection may mask the HCV infection or vice versa, resulting in difficulties of clinical diagnosis or furthermore complications during patients care due to analgesic toxicity. It has been considered that HCV can play a potential role in the hepatic disfunction related to the dengue infection [24]. Hepatic affectation related to chronic HCV infection can be a synergistic factor contributing to the increase of hemorrhagic cases during dengue infection, especially in adults [25].

Although there are no statistically significant differences, perinatal HCV infection is more common in girls than boys [26]. Other studies have suggested that sexual or drug abuse would be more associated with males [17].

It is known that, in endemic zones, patients come into adulthood with immunity to all the different DENV serotypes [27, 28]. HCV-DENV coinfections were presented mainly in young patients, so they are the ones who require more attention during the dengue outbreaks, since they may present severe complications.

It is well known that chronic infections affect platelet counting directly [18, 19] and HCV infection may lead to hemorrhagic problems [29]. In patients with HCV infection, where liver and the coagulation system are altered, clinical dengue manifestations could be grave.

Thus, HCV infection represents an important risk factor for complications in patients that also have dengue infection.

The HCV diagnosis must be substantially improved in the local health systems, primordially in dengue endemic areas, and the HCV testing for all those suspected acute dengue patients should be included for clinical management and prophylaxis.

## Figures and Tables

**Table 1:** Primers used for amplification in the molecular diagnosis for dengue and HCV.

Virus	Primer	Sequences	Fragment size (bp)	Reference
DENV	DV1 (+)	5′-GGRACKTCAGGWTCTCC-3′	—	[22]
	DV3 (−)	5′-AARTGIGCYTCRTCCAT-3′	470	
	DSP1 (−)	5′-AGTTTCTTTTCCTAAACACCTCG-3′	169	
	DSP2 (−)	5′-CCGGTGTGCTCRGCYCTGAT-3′	362	
	DSP3 (−)	5′-TTAGAGTYCTTAAGCGTCTCTTG-3′	265	
	DSP4 (−)	5′-CCTGGTTGATGACAAAAGTCTTG-3′	426	

HCV	HCV 1 (+)	5′-ACTCCACCATAGATCACTCCC-3′	241	[23]
	HCV 2 (−)	5′-AACACTACTCGGCTAGCAGT-3′		
	HCV 3 (+)	5′-TTCACGCAGAAAGCGTCTAG-3′	144	
	HCV 4 (−)	5′-CTTTATCCAAGAAAGGACCC-3′		

**Table 2:** Gender distribution of patients with dengue fever (DF) or hemorrhagic dengue (HD) and hepatitis C (HCV).

Gender	Dengue infected patients			Coinfected HCV-DENV patients		
	Total: 240 (100%)			Total: 31/240 (12.9%)		
	Total	DF	HD	Total	HCV-DF	HCV-HD
	*n* = 240	*n* = 120	*n* = 120	31	12/31	19/31
	(100%)	(50%)	(50%)	(100%)	(38.71%)	(61.29%)
Male	131 (100%)	66 (50.4%)	65 (49.6%)	16 (100%)	4/16 (25%)	12/16 (75%)
Female	109 (100%)	54 (49.5%)	55 (50.5%)	15 (100%)	8/15 (53.3%)	7/15 (46.7%)

**Table 3:** Age and gender distribution of coinfection HCV-dengue cases.

Age (years)	Male		Female	
	*n *	%	*n *	%
0–10	2	12.5	3	20
11–20	7	43.7	5	33.4
21–30	5	31.3	5	33.4
31–40	1	6.25	1	6.6
41–50	1	6.25	1	6.6
51–60	0	0	0	0
61–70	0	0	0	0
71–80	0	0	0	0

Total	16	100	15	100

Median	**23.5**		**23**	
±SD	21 ± 9		20 ± 12	

## References

[1] Lambrechts L, Scott TW, Gubler DJ (2010). Consequences of the expanding global distribution of aedes albopictus for dengue virus transmission. *PLoS Neglected Tropical Diseases*.

[2] Raheel U, Faheem M, Riaz MN (2011). Dengue fever in the Indian subcontinent: an overview. *The Journal of Infection in Developing Countries*.

[3] Halstead SB (2007). Dengue. *The Lancet*.

[4] WHO (2009). *Dengue: Guideline for Diagnosis, Treatment, Prevention and Control*.

[5] Malavige GN, Fernando S, Fernando DJ, Seneviratne SL (2004). Dengue viral infections. *Postgraduate Medical Journal*.

[6] Ramos C (2010). *La fiebre por dengue en México: un problema persistente de salud pública*.

[7] CENAVECE (2011). *Panorama Epidemiológico del Dengue*.

[8] Behera B, Chaudhry R, Pandey A (2009). Co-infections due to leptospira, dengue and hepatitis E: a diagnostic challenge. *The Journal of Infection in Developing Countries*.

[9] Bustos J, Hamdan A, Lorono MA, Montero MT, Gomez B (1990). Serologically proven acute rubella infection in patients with clinical diagnosis of dengue. *Epidemiology and Infection*.

[10] Zavala-Velázquez JE, Vado-Solís IA, Rodríguez-Félix ME (1998). Leptospirosis anictérica en un brote epidémico de dengue en la Península de Yucatán. *Revista Biomedica*.

[11] Loroño-Pino MA, Cropp CB, Farfán JA (1999). Common occurrence of concurrent infections by multiple dengue virus serotypes. *American Journal of Tropical Medicine and Hygiene*.

[12] Terzian ACB, Mondini A, Bronzoni RV (2011). Detection of Saint Louis Encephalitis virus in Dengue-suspected cases during a Dengue 3 outbreak. *Vector-Borne and Zoonotic Diseases*.

[13] Schwarz TF, Dobler G, Gilch S, Jager G (1994). Hepatitis C and arboviral antibodies in the island populations of Mauritius and Rodrigues. *Journal of Medical Virology*.

[14] Mohd Hanafiah K, Groeger J, Flaxman AD, Wiersma ST (2013). Global epidemiology of hepatitis C virus infection. New estimates of age-specific antibody to HCV seroprevalence. *Hepatology*.

[15] Lemon SM, Walker C, Alter MJ, Yi M, Howley DMK, PM DMK (2007). Hepatitis C virus. *Fields Virology*.

[16] Strauss JH, Strauss EG (2002). *Viruses and Human Diseases*.

[17] Burguete-Garcia AI, Conde-Gonzalez CJ, Jiménez-Mendez R (2011). Hepatitis C seroprevalence and correlation between viral load and viral genotype among primary care clients in Mexico. *Salud Pública de México*.

[18] Olariu M, Olariu C, Olteanu D (2010). Thrombocytopenia in chronic hepatitis C. *Journal of Gastrointestinal and Liver Diseases*.

[19] Aref S, Sleem T, Menshawy NE (2009). Antiplatelet antibodies contribute to thrombocytopenia associated with chronic hepatitis C virus infection. *Hematology*.

[20] Arya SC, Agarwal N (2011). Thrombocytopenia progression in dengue cases during the 2010 outbreak in Indian capital metropolis. *Platelets*.

[21] Loroño-Pino MA, Farfán-Ale JA, Zapata-Peraza AL (2004). Introduction of the American/Asian genotype of dengue 2 virus into the Yucatan State of Mexico. *American Journal of Tropical Medicine and Hygiene*.

[22] Seah CLK, Chow VTK, Tan HC, Chan YC (1995). Rapid, single-step RT-PCR typing of dengue viruses using five NS3 gene primers. *Journal of Virological Methods*.

[23] Okamoto H, Okada S, Sugiyama Y (1990). Detection of hepatitis C virus RNA by a two-stage polymerase chain reaction with two pairs of primers deduced from the 5′-noncoding region. *Japanese Journal of Experimental Medicine*.

[24] Trung DT, Thao LTT, Hien TT (2010). Liver involvement associated with dengue infection in adults in Vietnam. *American Journal of Tropical Medicine and Hygiene*.

[25] Abdul Wahid SFS, Sanusi S, Zawawi MM, Ali RA (2000). A comparison of the pattern of liver involvement in dengue hemorrhagic fever with classic dengue fever. *Southeast Asian Journal of Tropical Medicine and Public Health*.

[26] Madurga Revilla P, Aguar Carrascosa M, Pereda Perez A (2012). Retrospective study of risk factors of vertical transmission of hepatitis C virus. *Anales de Pediatría*.

[27] Muhammad Azami NA, Salleh S, Neoh H, Syed Zakaria S, Jamal R (2011). Dengue epidemic in Malaysia: not a predominantly urban disease anymore. *BMC Research Notes*.

[28] Thai KTD, Nishiura H, Hoang PL (2011). Age-specificity of clinical dengue during primary and secondary infections. *PLoS Neglected Tropical Diseases*.

[29] Morano CM, Perrone A, Deramo MT, Santarangelo P, Schiraldi O (2000). Hepatitis C virus infection mimicking hemorrhagic fever: a case report. *Infezioni in Medicina*.

